# ^1^H, ^13^C, and ^15^N backbone resonance assignment of the N-terminal 280 amino acids of human NHERF1 protein

**DOI:** 10.1007/s12104-026-10281-6

**Published:** 2026-10-29

**Authors:** Almudena Hornero-Romera, Miguel Á. Treviño, David Pantoja-Uceda, Jaime Millán, M. Ángeles Jiménez

**Affiliations:** 1 Instituto de Química Física Blas Cabrera (IQF), CSIC, Madrid, Spain; 2 Centro de Biología Molecular Severo Ochoa (CBM), CSIC-UAM, Madrid, Spain

**Keywords:** NMR, PDZ domain, NHERF

## Abstract

NHERF1 (Na+/H+ exchanger regulatory factor-1) is a membrane-associated scaffold protein that links membrane receptors and channels to the actin cytoskeleton. It plays key roles in protein trafficking, signaling, and liver physiology. NHERF1 is a 358-residue long multidomain protein consisting of two PDZ domains, followed by a largely disordered region and a C-terminal ezrin-binding (EB) domain. In the liver, NHERF1 promotes the assembly of complexes containing ERM (ezrin-radixin-moesin) proteins, ICAM-1 (type I transmembrane protein intercellular adhesion molecule), and F-actin. Recent pull-down experiments have suggested that both PDZ1 and PDZ2 domains contribute to ICAM-1 binding. Characterization of this interaction by NMR chemical shift mapping requires a construct containing both PDZ domains. Although NMR studies have previously been reported for the individual PDZ1 and PDZ2 domains, as well as for the full-length NHERF1, no assignments were available for a construct containing only the PDZ domains. Full-length NHERF1 is not adequate for investigating PDZ-mediated interactions because its C-terminal EB domain interacts with PDZ2, causing resonance line broadening. In addition, the full-length 358-residue protein is expected to exhibit greater spectral overlap than a shorter construct. Here, we report the ^1^H, ^15^N, and ^13^C backbone resonance assignments of a N-terminal 280 residue long NHERF1 construct encompassing both the PDZ1 and PDZ2 domains, thereby facilitating future studies of its interactions with ICAM-1 and other binding partners.

**Supplementary Information:**

The online version contains supplementary material available at https://doi.org/10.1007/s12104-026-10281-6.

## Biological context

**Fig. 1:**
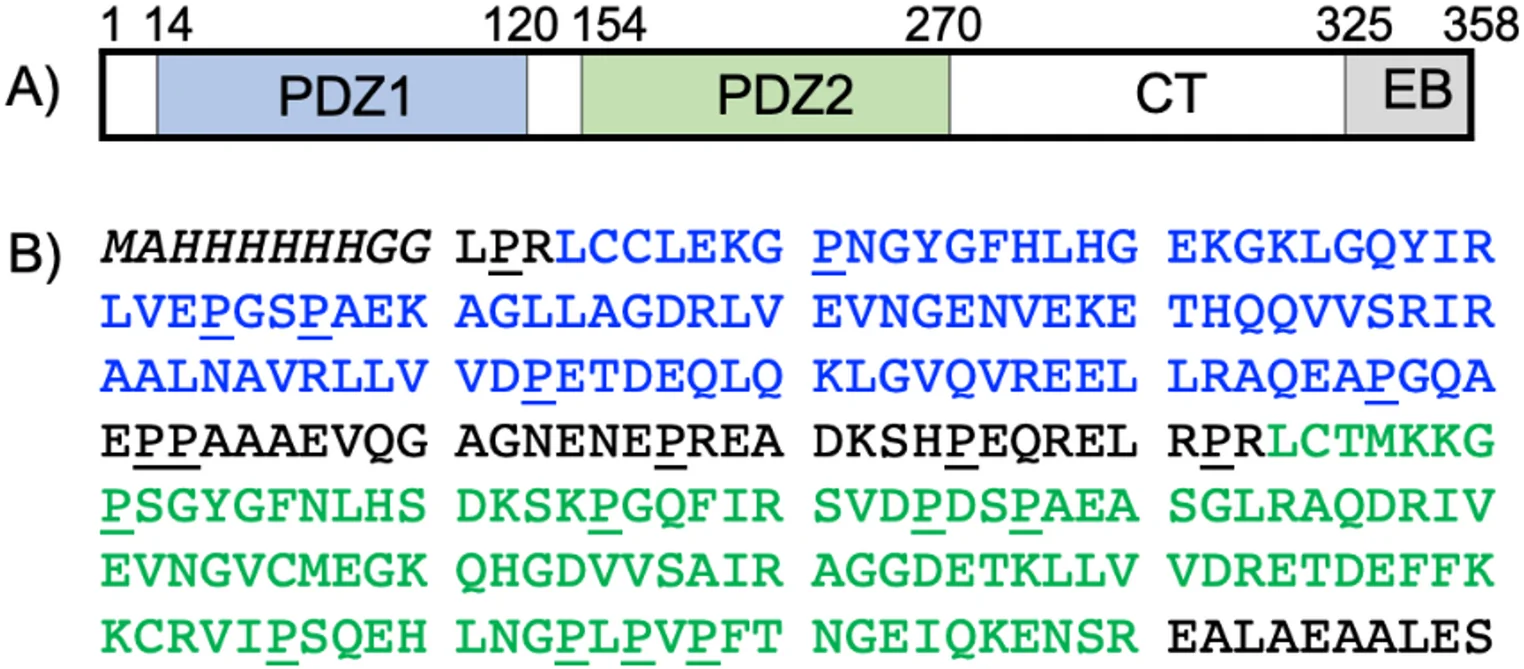
(**A**) Schematic diagram of human NHERF1 domains. (**B**) Sequence of NHERF1 ^(11–280)^. Cloning tag residues are in italics. PDZ1 and PDZ2 residues are in blue and green, respectively. Prolines are underlined

Na^+^/H^+^ exchanger regulatory factor (NHERF1) is an adaptor protein, densely distributed in the apical membranes of polarized epithelial cells in numerous mammalian tissues (Hall et al. 1998). NHERF1 participates in organizing the trafficking, localization, and membrane targeting of a large variety of membrane receptors and channels through direct binding interactions (Hall et al. 1998). As a multidomain and multivalent protein, NHERF1 recruits different signaling partners. NHERF1 consists of two PDZ domains, followed by a long mostly disordered region (CT) and an ezrin binding (EB) domain (Fig. 1A). The NHERF1 PDZ domains bind to the consensus PDZ-binding motif D(S/T)X(V/I/L) (where X denotes any amino acid residue) located at the carboxyl termini of a number of membrane channels or receptors (Karthikeyan et al. 2001). The carboxyl terminus of NHERF1 recognizes the FERM domain, which is a conserved domain shared by protein 4.1, ezrin, radixin, and moesin, proteins involved in cytoskeletal actin-binding (Li et al. 2005). Thus, NHERF1 is responsible for bridging the interactions between cell membrane and the cytoskeletal actin networks. As a membrane cytoskeleton adapter protein, NHERF1 enhances the efficiency and specificity of membrane processes by forming multiprotein macromolecular complexes (Shenolikar et al. 2004). In mouse liver, NHERF1 assembles ERM proteins, ICAM-1 (type I transmembrane protein intercellular adhesion molecule) and F-actin into a macromolecular complex. Deletion of NHERF1 in mice leads to reduced expression of hepatic ERM proteins and ICAM-1 molecules that are up-regulated and are essential for neutrophil migration and infiltration after bile duct ligation (BDL) (Li et al. 2015), a surgical procedure commonly used in animal models to study liver diseases. NHERF1 deficiency also increased synthesis of tetrahydroxylated bile acid, which can suppress bile acid-induced liver damage. Together, these mechanisms protect NHERF1 -/- mice against cholestatic liver injury induced by BDL (Li et al. 2015).

Pull-down experiments suggested that both the PDZ1 and PDZ2 domains of NHERF1 are involved in the interactions with ICAM-1 (Cacho-Navas et al. 2024). Identification of the ICAM-1 binding site within these domains could be achieved through chemical shift mapping using an NHERF1 construct comprising both PDZ domains. Although NMR studies have been reported for constructs containing either the PDZ1 or the PDZ2 domain (Bhattacharya et al. 2013), as well as for the full-length NHERF1 (Bhattacharya et al. 2019), no resonance assignments are available for a construct comprising only the two PDZ domains. To examine interactions across both PDZ domains, the full-length NHERF1 protein is not optimal because its C-terminal EB domain interacts intramolecularly with PDZ2 (Centonze et al. 2018), resulting in substantial line broadening of many PDZ2 resonances and of some PDZ1 resonances (Bhattacharya et al. 2019). Furthermore, spectral overlap is expected to be more severe in the full-length 358-residue protein than in a shorter construct containing only the PDZ1 and PDZ2 domains. To address these limitations, we report the ^1^H, ^15^N, and ^13^C backbone resonance assignments of a 280-residue NHERF1 construct encompassing the PDZ1 and PDZ2 domains (NHERF1^(11–280)^; Fig. 1B). These assignments provide a basis for future studies of its interactions with ICAM-1 and other binding partners.

## Methods and experiments

### Protein expression and purification

The codifying sequence for 11 to 280 residues of NHERF1 was cloned in a pET24(+) vector, which added a His_6_ tag in N terminal position of the construct (Fig. 1B), and was transformed in E. coli BL21star(DE3) bacteria. The uniformly ^13^C,^15^N-labelled protein was expressed following the protocol previously described (Treviño 2025). Briefly, cells were grown at 25 °C for 20–24 h in minimal medium M9 + + containing limited quantities of nutrients (0.06% ^15^NH_4_Cl and 0.2% glucose) to ensure their complete consumption. More nutrients were then added (0.3% ^15^NH_4_Cl and 1% ^13^C-glucose for ^15^N-^13^C labelling) alongside 0.5 mM IPTG to induce protein expression. After overnight expression at 25 °C the cells were centrifuged, lysed by sonication and purified by chromatography using a 5mL Ni-Charged Resin FF Prepacked Column (GenScipt, Piscataway, NJ, USA), followed by dialysis and a second chromatographic step through a 5mL HiTrapQ column (Cytiva Marlborough, MA, USA). The ^13^C,^15^N-NHERF1^(11–280)^ protein was dialyzed in 5mM potassium phosphate (pH 6.4), 10mM NaCl and 2mM β-mercaptoethanol and concentrated by centrifugal ultrafiltration to 0.8-0-9mM in a vivaspin 20 with a 10 kDa cutoff membrane (Sartorius, Göttingen, Germany). A volume of 450 µl of this dialyzed protein plus 50 µl D_2_O (10% D_2_O for lock) was placed in a 5 mm NMR tube and used for acquisition of NMR spectra.

### NMR spectroscopy

NMR experiments were performed on a Bruker Avance NEO spectrometer operating at 600 MHz, equipped with a QCI cryogenic probe and z-axis gradients. All data were acquired at 298 K and processed using TopSpin 4.4. Backbone resonance assignments were obtained from standard triple-resonance experiments: 2D ^1^H–^15^N HSQC and 3D HNCO, HNcaCO, HNCA, CBCAcoNH, and HNCACB (Sattler et al. 1999). Additionally, the ^15^N spectral width was optimized using an in-house program, which allowed a 25% reduction in spectral resolution without compromising assignment quality. For further details on acquisition parameters, please refer to the Supplementary Table S1. Chemical shifts were referenced to DSS for ^1^H and indirectly for ^13^C and ^15^N according to IUPAC recommendations (Wishart et al. 1995). Assignments were performed manually using CcpNmr Analysis Assign (Skinner et al. 2016).

## Extent of assignment and data deposition

### Backbone resonance assignment

**Fig. 2:**
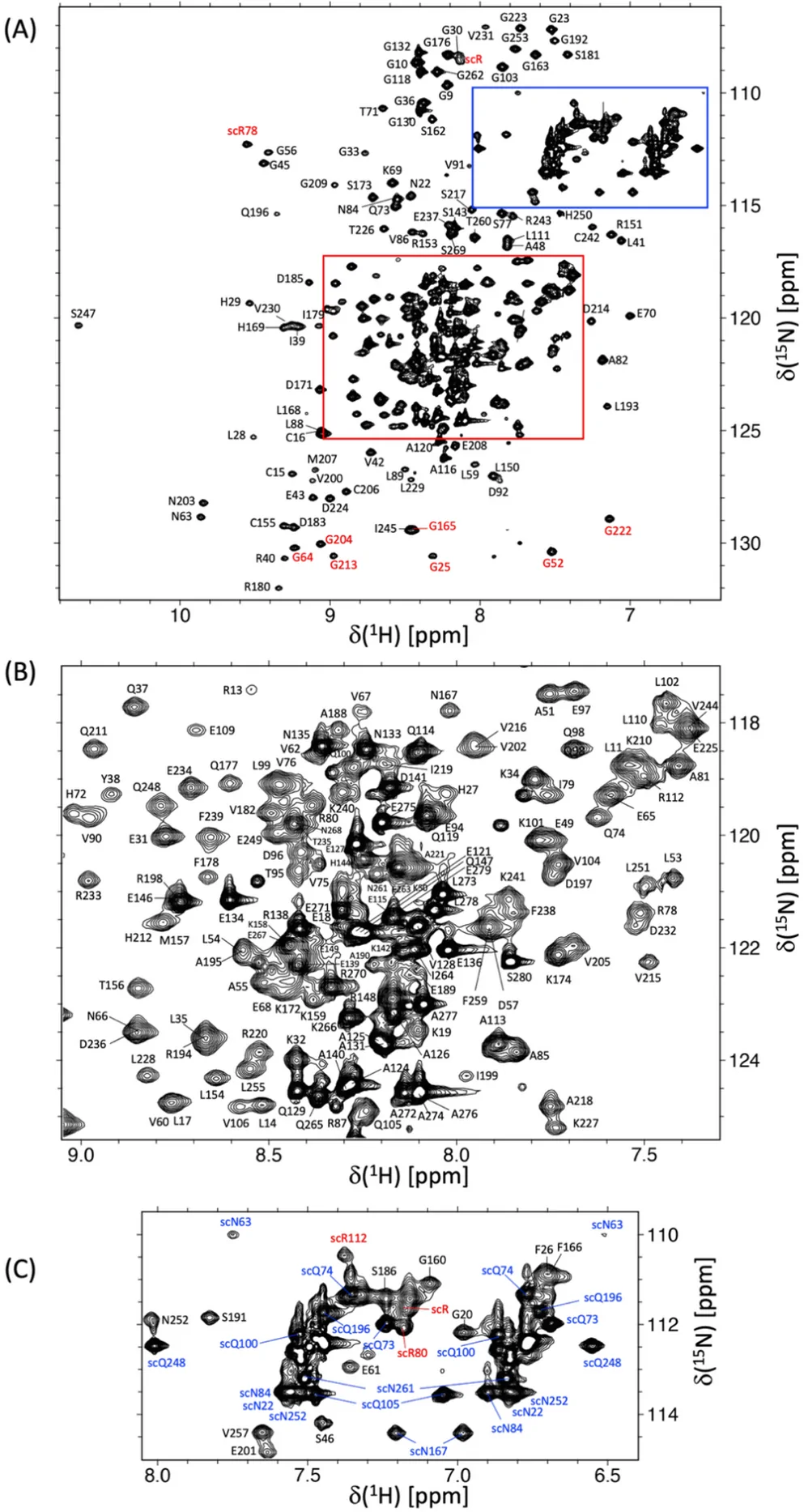
^1^H–^15^N HSQC spectrum of ^13^C,^15^N- NHERF1^(11–280)^ in aqueous solution at pH 6.4 and 25 °C. The full spectrum (**A)** is shown together with expansions of the two boxed regions: (**B**) the crowded central region, and (**C**) region containing Asn and Gln sidechains. The assignments of the backbone amide cross-peaks are indicated by one-letter residue type and sequence number. In the case of cross-peaks belonging to Arg, Asn and Gln side chains, “sc” precedes to one-letter residue type and sequence number. Arg side chains and some Gly residues, which were folded, are labelled in red. Asn and Gln side chains are labelled in blue

The good dispersion of cross-peaks in the ^1^H–^15^N HSQC of NHERF1^(11–280)^ (Fig. 2A) indicates that the protein is well-folded.

Backbone ^1^H_N_, ^15^N, ^13^C’, ^13^Cα and ^13^C_β_ chemical shifts were assigned through the analysis of a series of triple resonance 3D NMR experiments (HNCO, HNcaCO, HNCA, CBCAcoNH, HNCACB). Excluding the cloning tag (Fig. 1B), the ^1^H_N_ and ^15^N chemical shifts were assigned for a total of 244 of the 251 observable residues (97.2%). The non-Pro ^1^H-^15^N cross-peaks, which could not be assigned, correspond to Y24, R58, L83, R107, and E108 in PDZ1 and Y164 and S170 in PDZ2, being R107 the only residue for which no ^13^C nuclei was assigned. Concerning the ^13^C chemical shifts, assignment completeness was 90.7% for ^13^Cα (245/270), 98.8% for ^13^Cβ (241/244) and 90.7% for ^13^C’ (245/270). In addition, ^1^H-^15^N cross-peaks for three residues of the cloning tag (A2, G9, G10) were identified. Moreover, the ^1^H, ^15^N and ^13^C chemical shifts of the side-chain amide groups of six Asn (N22, N63, N84, N167, N252, N261) and nine Gln (Q73, Q74, Q98, Q100, Q105, Q129, Q196, Q211, Q248) were unambiguously assigned based on cross-peaks observed in the 3D experiments.

^13^C_β_ chemical shifts were assigned for all Pro residues, except for the Pro-preceding Pro122, whose assignment was not possible using the set of 3D experiments analyzed. The assigned ^13^C_β_ chemical shifts for Pro residues are in the range 30.65–32.86 ppm, indicating that all of them are in the *trans* configuration (Schubert et al. 2002).

Regarding the redox state of the five Cys residues, all were found to be in the reduced form based on their ^13^C_β_ chemical shifts, which are in the range 27.12 to 29.92 ppm (Sharma and Rajarathnam 2000).

### Secondary structure and dynamical properties derived from chemical shifts

**Fig. 3:**
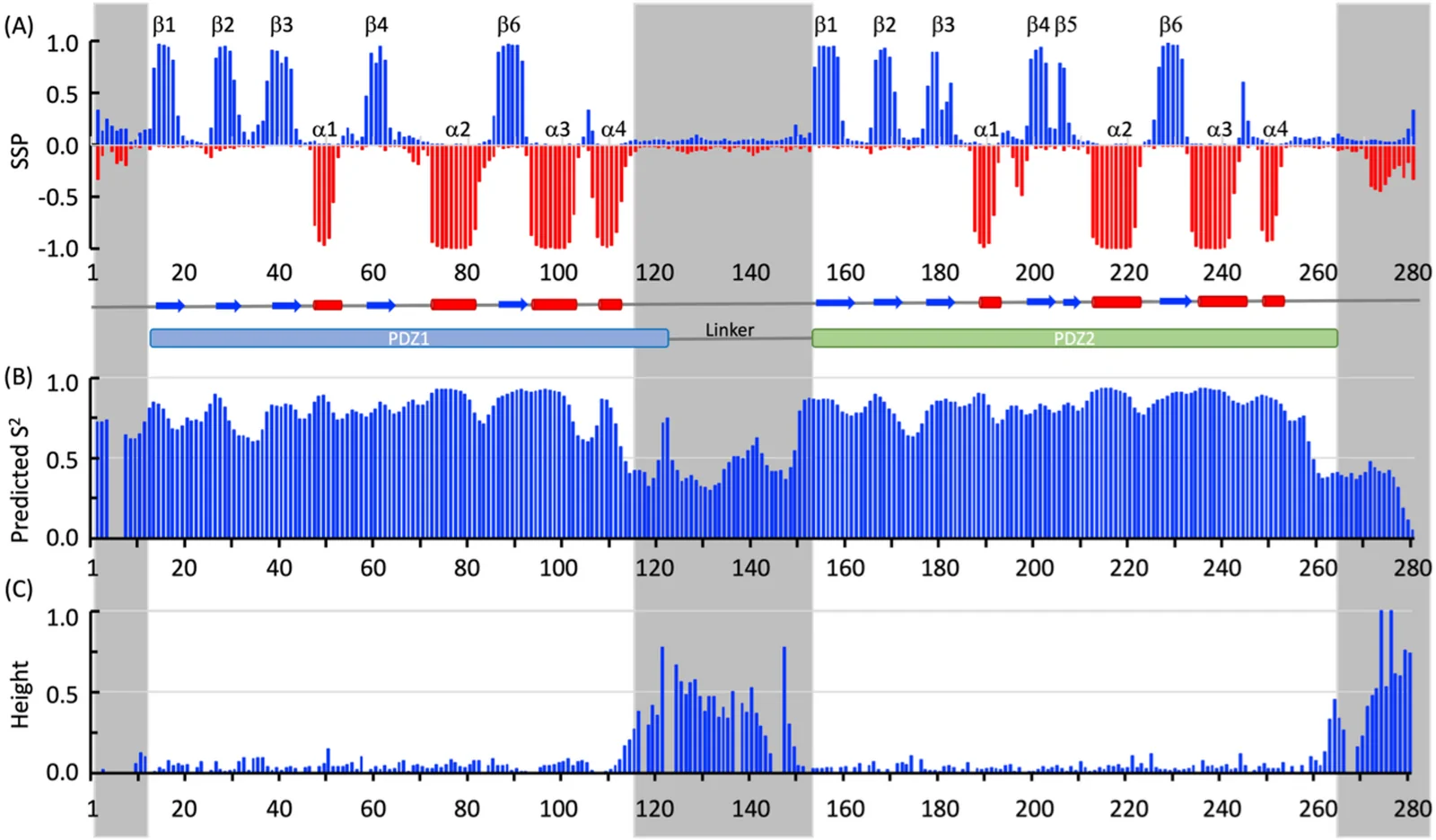
(**A**) Chemical shift derived secondary structure (SSP) for NHERF1^(11–280)^ using TALOS-N server. SSP confidence values for strand (blue bars) and helix (red bars) are plotted as a function of residue number. Strands (blue arrows) and helices (red cylinders) deduced from the confidence values. (**B**) Chemical shift predicted S^2^ order parameter for NHERF1^(11–280)^ plotted as a function of residue number. (**C)** Cross-peaks heights in ^1^H–^15^N HSQC spectrum of NHERF1^(11–280)^ plotted as a function of residue number. In all panels, the N-terminal tag, the linker region and the C-terminal region are surrounded by vertical grey rectangles

Secondary structure was derived from the ^1^H_N_, ^15^N, ^13^C’, ^13^C_α_ and ^13^C_β_ chemical shifts assigned for NHERF1^(11–280)^ by using the TALOS-N web server (Shen & Bax, 2013). As shown in Fig. 2A, the regions corresponding to PDZ1 and PDZ2 domains show 5–6 β-strands and 4 α-helices arranged in a β1-β2-β3-α1-β4-β5-α2-β6-α3-α4 topology, which is characteristic of PDZ domains. In contrast, the N-terminal cloning-tag, the linker, and the C-terminal region exhibit no clear tendency to adopt any secondary structure. This result is consistent with the AlphaFold 2 (AF2) model of the full-length NHERF1 protein (358 residues long), and the AlphaFold 3 (AF3; Supplementary Fig. S1) prediction for NHERF1^(11–280)^. The β-strands and α-helices span approximately the same residues that are predicted by AlphaFold and present in the NMR structures of the isolated PDZ1 and PDZ2 domains (2M0T & 2KJD; Supplementary Table S2). The main difference is that the PDZ1 domain in the NHERF1 ^(11–280)^ construct does not present the β5 strand (Fig. 3A & Supplementary Table S2).

Besides secondary structure, the order parameter S^2^, which reflects the local rigidity or flexibility of individual residues, can be predicted from chemical shifts (Berjanskii and Wishart 2005). Figure 3B shows the chemical shift-predicted S^2^ values for the NHERF1^(11–280)^ construct. The linker and C-terminal regions display lower chemical shift-predicted S^2^ values than the PDZ1 and PDZ2 domains. This indicates the linker and C-terminal regions likely to be highly flexible, whereas the PDZ domains behave as rigid structural units. This information is consistent with the analysis of the cross-peaks’ heights in the ^1^H–^15^N HSQC spectrum (Fig. 3C). Due to transverse T_2_ relaxation times, cross-peaks from rigid residues are broader, and consequently less high, than those from flexible residues. In the case of NHERF1^(11–280)^, the ^1^H–^15^N HSQC cross-peaks corresponding to residues within the linker and the C-terminal region are narrower, and so higher, than those arising from residues in the PDZ domains (Fig. 3C). Thus, the regions with well-defined secondary structures are more rigid than those with no clear secondary structure tendencies. In conclusion, the availability of the ^1^H, ^15^N, and ^13^C backbone resonance assignments of NHERF1^(11-280)^ provide a foundation for future investigations of its interactions with ICAM-1 and other binding partners.

## Supplementary Information

Below is the link to the electronic supplementary material.

Supplementary Material 1

## Data Availability

Chemical shifts have been deposited to the Biological Magnetic Resonance Data Bank (BMRB, http://bmrb.io) under accession number 53788.
