# Supplementary material for: 1H, 13C, and 15N backbone resonance assignment of the N-terminal 280 amino acids of human NHERF1 protein
Source: Biomol NMR Assign. 2026 Oct 29;20(1):31. doi: 10.1007/s12104-026-10281-6 (PMC13719848; doi:10.1007/s12104-026-10281-6)
Supplement: Supplementary file 1 [file 12104_2026_10281_MOESM1_ESM.docx]

**Supplementary information**

**for**

**^1^H, ^13^C, and ^15^N backbone resonance assignment of the N-terminal 280 amino acids of human NHERF1 protein**

Almudena Hornero-Romera,^1^ Miguel Á. Treviño,^1^ David Pantoja-Uceda,^1^ Jaime Millán,^2^ M. Ángeles Jiménez^1^*

1. Instituto de Química Física Blas Cabrera (IQF), CSIC, Madrid, Spain

2. Centro de Biología Molecular Severo Ochoa (CBM), CSIC-UAM, Madrid, Spain

**List of tables:**

**Table S1.** Relevant NMR acquisition parameters.

**Table S2.** Secondary structure elements deduced from chemical shifts for NHERF1^(11-280)^ (this work), present in experimental 3D structures for isolated PDZ1 and PDZ2 domains, and in the AlphaFold 2 model for the full-length NHERF1 protein.

**List of figures:**

**Figure S1.** Ribbon representation of the AlphaFold (AF3) model for the 11-280 region of NHERF1.

**Table S1:** Relevant NMR acquisition parameters.

| Experiment | TD  (points) | Spectral width  (ppm) | Center  (ppm) | FIDRES  (Hz) | Nº scans | Acquisition time |
| --- | --- | --- | --- | --- | --- | --- |
| ^1^H–^15^N HSQC | (^1^H) 2k  (^15^N) 128 | 13.6  35.0 | 4.7  119.0 | 8.0  33.26 | 4 | 10 min |
| HNCO | (^1^H) 2k  (^15^N) 64  (^13^C) 90 | 13.6  26.9  14.0 | 4.7  119.547  173.5 | 8.0  25.56  46.96 | 8 | 16 h |
| HNcaCO | (^1^H) 2k  (^15^N) 64  (^13^C) 90 | 13.6  26.9  14.0 | 4.7  119.547  173.5 | 8.0  25.56  46.96 | 8 | 16 h |
| HNCA | (^1^H) 2k  (^15^N) 64  (^13^C) 128 | 13.6  26.9  30.0 | 4.7  119, 547  53.2 | 8.0  25.56  70.75 | 8 | 22.0 h |
| CBCAcoNH | (^1^H) 2k  (^15^N) 64  (^13^C) 128 | 13.6  26.9  80.0 | 4.7  119. 547  43.0 | 8.0  25.56  188.65 | 16 | 45.0 h |
| HNCACB | (^1^H) 2k  (^15^N) 64  (^13^C) 128 | 13.6  26.9  80.0 | 4.7  119.547  43.0 | 8,0  25.56  188.65 | 40 | 112 h |

**Table S2**. Secondary structure elements deduced from chemical shifts for NHERF1^(11-280)^ (this work), present in experimental 3D structures for isolated PDZ1 and PDZ2 domains, and in the AlphaFold 2 (AF2) model for the full-length NHERF1 protein.

|  | PDZ1 | | | PDZ2 | | |
| --- | --- | --- | --- | --- | --- | --- |
| Secondary structure | This work | 2M0T | AF2 | This work | 2KJD | AF2 |
| β1 | 13-17 | 13-18 | 13-18 | 153-158 | 153-158 | 153-158 |
| β2 | 26-30 | 26-30 | 26-29 | 155-170 | 166-170 | 166-169 |
| β3 | 37-42 | 37-42 | 37-42 | 177-182 | 177-182 | 177-182 |
| α1 | 47-51 | 46-52 | 47-49 | 187-191 | 186-192 | 187-190 |
| β4 | 59-62 | 58-62 | 58-62 | 200-202 | 198-202 | 198-202 |
| β5 | --- | 65-66 | 65-66 | 205-206 | 205-206 | 205-206 |
| α2 | 72-81 | 71-80 | 72-81 | 212-221 | 211-221 | 212-221 |
| β6 | 86-91 | 85-91 | 85-91 | 226-231 | 225-231 | 225-231 |
| α3 | 93-102 | 92-101 | 93-102 | 233-241 | 232-243 | 233-241 |
| α4 | 106-112 | 108-113 | 108-112 | 248-251 | 247-252 | 248-251 |

**Figure S1.** Ribbon representation of the AlphaFold (AF2) model for the N-terminal region of NHERF1. PDZ1 and PDZ2 domains are, respectively, in light-blue and light-green backgrounds. N- and C-terminal ends, β-strands and α-helices are labelled.
